# Exploratory serum biomarker profiling in idiopathic REM sleep behavior disorder: evidence of early neurodegenerative signatures: a pilot study

**DOI:** 10.1007/s11325-026-03698-9

**Published:** 2026-05-11

**Authors:** Danielle Mesquita Torres, Fábio Henrique Queiroz Pereira, Marylane da Silva Viana, Mariana Michilles Santos Ramos, Elisa Gabriela Barros Cunha, Leonardo José Rodrigues de Araújo Melo, Emmanuelle Silva Tavares Sobreira, Francisca Charliane Almino Lopes, Annyta Fernandes Frota, Michelle Verde Ramos Soares, Ricardo Titze- de- Almeida, Pedro Braga-Neto, Danielle S. Macedo, Manoel Alves Sobreira-Neto

**Affiliations:** 1 Postgraduate Program in Translational Medicine, Universidade Federal do Ceará, Fortaleza, Brazil; 2 Department of Physiology and Pharmacology, Neuropsychopharmacology Laboratory, Drug Research and Development Center, Faculty of Medicine, Universidade Federal do Ceará, Fortaleza, CE Brazil; 3 Faculty of Medicine, Division of Neurology, Universidade Federal do Ceará, Fortaleza, CE Brazil; 4 Universidade de Brasília, Brasília, DF Brazil; 5 Clinical Medicine Departament, Faculty of Medicine of Federal, University of Ceará, Rua Prof. Costa Mendes, 1408 – 4º. Andar, Fortaleza, CEP: 60.430-140 Brazil; 6 Department of Translational Medicine, Federal University of Ceará, Rua Coronel Nunes de Melo, n 1000, Fortaleza, CEP 60.430-275 Brazil

**Keywords:** REM sleep behavior disorder, Synucleinopathies, Neurodegeneration, Biomarker

## Abstract

**Background:**

Idiopathic REM sleep behavior disorder (iRBD) indicates an early α-synucleinopathy, offering a window for early detection and intervention.

**Objective:**

Explore a serum-based biomarker panel, comprising NfL, GFAP, t-tau, and UCH-L1, in patients with iRBD, and compare to individuals with synucleinopathy and healthy controls.

**Method:**

This is a cross-sectional, observational study. Serum biomarkers were measured using the single molecule array technique. Clinical evaluation included standardized assessments of sleep, cognitive function, motor symptoms, and psychiatric features.

**Results:**

GFAP levels were significantly higher in the iRBD group than in the control and synucleinopathy groups. Serum NfL levels yielded conflicting results. T-tau and UCHL-1 protein levels were lower in the iRBD and synucleinopathy groups compared to the control group.

**Conclusion:**

Our findings suggest that serum levels of NfL, GFAP, t-tau, and UCH-L1 may serve as potential biomarkers in iRBD, primarily GFAP levels.

## Introduction

REM sleep behavior disorder (RBD) is a parasomnia characterized by dream enactment, with loss of physiological muscle atonia during REM sleep, and by vocal and/or motor behaviors [1]. Idiopathic RBD (iRBD) indicates an early α-synucleinopathy, with approximately 90% of individuals developing clinically manifest Parkinson’s disease (PD), dementia with Lewy Bodies (DLB), or multiple system atrophy (MSA) within 15 years of iRBD diagnosis [2, 3].

A broad definition of a biomarker is an indicator of normal biological processes, pathogenic processes, or responses to an exposure or intervention [4].They could be helpful not only for diagnosis but also for therapeutic management and monitoring of iRBD progression [5]. Serum biomarkers are minimally invasive, clinically accessible tools with growing potential to predict phenoconversion [6, 7]. Research is currently underway to assess the biomarker potential of miRNAs obtained from peripheral blood samples [8, 9]. Research on plasma biomarkers has also analyzed the potential of neurofilament (Nfl), glial fibrillary acid protein (GFAP), ubiquitin C-terminal hydrolase L1 (UCH-L1), and Total tau (t-tau), neurodegeneration markers, as biomarkers in PD, using the single-molecule array (SIMOA) platform [10]. However, its applicability in assessing phenoconversion in patients with iRBD remains undetermined.

The present study aimed to explore a serum-based biomarker panel, comprising Nfl, GFAP, t-tau, and UCH-L1, in patients with iRBD and to compare these profiles with those observed in individuals with established synucleinopathy and healthy controls. This multi-analyte approach may offer additional pathophysiological insight and discriminatory power, potentially enhancing early risk stratification in the prodromal phase of synucleinopathies.

## Methods

Participants were recruited from the community and from the Sleep and Parkinson’s disease outpatient clinics at the University Hospital of the Federal University of Ceará (HU-UFC). PD diagnosis followed the Movement Disorder Society clinical diagnostic criteria [11]. The diagnosis of RBD was established in accordance with the International Classification of Sleep Disorders, Third Edition (ICSD-3) and the American Academy of Sleep Medicine (AASM) polysomnographic criteria [12, 13]. Patients who were using antidepressants had their medications discontinued to assess causality, establishing the diagnosis of antidepressant-related RBD. A healthy control group was recruited from the community, matched for age and sex, and included unrelated individuals with no family history linking them to the patients. RSWA (REM sleep without atonia) quantification during v-PSG (video polysomnography) was performed according to the cut-offs established by the SINBAR group study [14].

Clinical evaluation was performed using the following scales: Pittsburgh Sleep Quality Index (PSQI), Epworth Sleep Scale (ESS), Stop Bang score, Beck’s Depression Inventory (BDI), Beck Anxiety Inventory (BAI), Mini-Mental Status Examination (MMSE), Montreal Cognitive Assessment (MoCA), and Movement Disorder Society-Unified Parkinson’s Disease Rating Scale (MDS-UPDRS-III). After, the participants underwent blood collection and v-PSG. Serum biomarkers were measured by Simoa technique on the Simoa SR-X Analyzer (Quanterix, Lexington, MA) as established previously [15]. The lower limits of quantification (LLOQ) for each biomarker were as follows: 0.276 pg/mL for GFAP, 0.136 pg/mL for NfL, 4.03 pg/mL for UCH-L1, and 0.0298 pg/mL for t-tau. The Nfl values ​​were adjusted for age and body mass index (BMI) using the swiss national reference database and web application [16]. V-PSG was performed using a digital polygraph in accordance with AASM guidelines.

This study was approved by the Ethics Committee of HU-UFC, protocol number (CAAE 74817423.8.10015045). The study was approved according to the principles of the Declaration of Helsinki.

The normality of the data distribution was assessed using the Kolmogorov–Smirnov test. Because most variables did not follow a normal distribution, nonparametric tests were initially used. Correlations among variables were evaluated using Spearman’s correlation. Data were processed and analyzed using Microsoft Excel and R software version 4.1. Given the nature of the data and the presence of groups with small and unequal sample sizes, particularly the iRBD group (*n* = 4), the robustness of the inferences was ensured by applying the simple Bootstrap method with 1000 resamples, using bias-corrected and accelerated confidence intervals (BCa) at the 95% level. One-way ANOVA and post hoc analysis were also conducted. The datasets analyzed during the current study are available from the corresponding author on reasonable request.

## Results

A total of 31 participants were included in our study, divided into four groups (control, iRBD, antidepressant-related RBD, and PD). 11 patients were enrolled in the control group. 4 patients were diagnosed with iRBD, 14 with PD, and only 2 participants with antidepressant-related RBD (Table 1). These last ones, based on a small sample, were removed from the statistical analyses. The groups did not differ in terms of sex or age.

**Table 1:** Comparison between socio-demographic data, standardized scales scores and serum biomarkers levels of controls, iRBD and PD patients (*n* = 29)

	Control (*n* = 11)	iRBD (*n* = 4)	RBD + PD (*n* = 14)	*p*
Age (years), mean ± SD	64 ± 15	66 ± 3	67 ± 7	0.966
Male sex, n (%)	5 (45%)	3 (75%)	6 (43%)	
PSQI, mean ± SD	N/A	16 ± 7.8	14.8 ± 7.3	0.955
ESS, mean ± SD	N/A	10.5 ± 1.7	12.5 ± 5	0.331
SBQ, mean ± SD	N/A	3.25 ± 0.96	3.62 ± 0.77	0.494
MMSE, mean ± SD	N/A	29 ± 0.8	24.1 ± 5	0.040*
MoCA, mean ± SD	N/A	24 ± 2	19 ± 8	0.460
BDI, mean ± SD	N/A	8 ± 4	17 ± 15	0.364
BAI, mean ± SD	N/A	19 ± 15	18 ± 15	0.692
GFAP, mean ± SD	203 ± 111	474 ± 132	265 ± 147	0.019*
NfL, mean ± SD	15 ± 8	25 ± 7	28 ± 15	0.026*
NfL z-score mean ± SD	0.35 ± 1.3	1.86 ± 0.47	1.76 ± 1.07	0.019*
NfL percentile mean ± SD	56 ± 35	96 ± 3	87 ± 22	0.020*
T-tau, mean ± SD	8.26 ± 1.97	0.79 ± 0.37	1.08 ± 0.47	< 0.001*
UCH-L1, mean ± SD	52 ± 18	8 ± 7	9 ± 4	< 0.001*
UPDRS-III, mean ± SD	N/A	0.25 ± 0.5	40.31 ± 21.6	< 0.001*

*iRBD* Idiopathic REM Sleep Behavior Disorder, *PD* Parkinson’s Disease, *SD* Standard Deviation, *PSQI* Pittsburgh Sleep Quality Index, *ESS *Epworth Sleepiness Scale, *SBQ* STOP-Bang Questionnaire, *MMSE* Mini Mental State Examination, *MoCA* Montreal Cognitive Assessment, *BDI* Beck Depression Inventory, *BAI* Beck Anxiety Inventory, *GFAP* Glial Fibrillary Acidic Protein, *NfL* Neurofilament, *T-tau* Tau Protein, *UCH-L1* Ubiquitin C-terminal Hydrolase L1, *N/A* Not Available. (*P < 0.05). Units: GFAP ng/ml, Nfl pg/ml, Nfl z-score pg/ml, Nfl precentile pg/ml, T-tau pg/ml, UCH-L1 pg/ml

Regarding the cognitive, mood disorder, and sleep quality scales, we found a difference only between the iRBD and PD groups on the MMSE (*p* = 0.04). The PD group obtained the lowest score. The median UPDRS-III score in the PD group was significantly higher (*p* < 0.001) (Table 1).

**Fig. 1:**
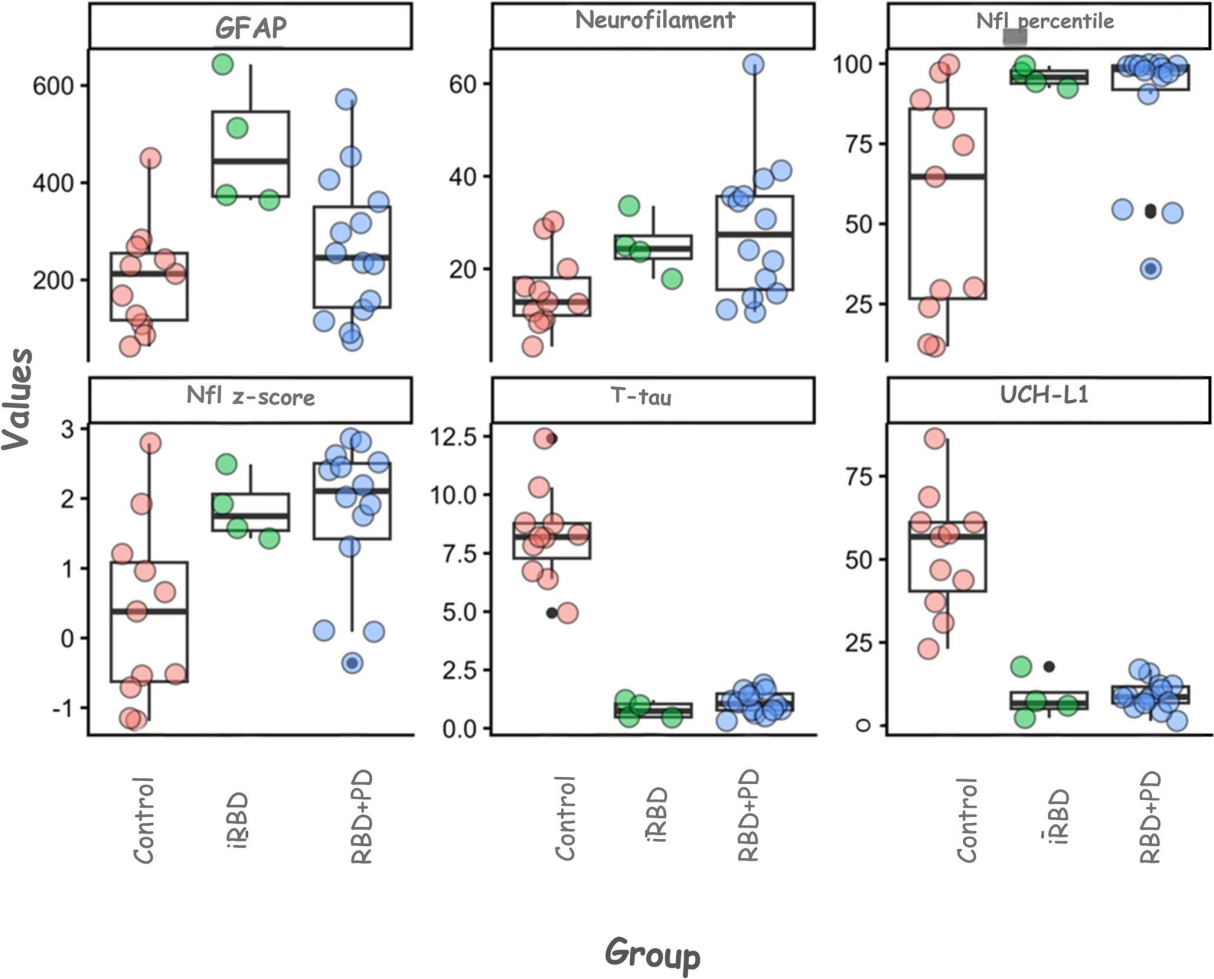
Serum levels of GFAP, neurofilament light chain (NfL), T-tau, and UCH-L1 across study groups. Biomarker concentrations were measured using the Simoa SR-X platform in three groups: healthy controls (red), patients with idiopathic REM sleep behavior disorder (iRBD, green), and patients with established synucleinopathy (blue). Data are represented as individual values overlaid on box plots showing the median and interquartile range. Statistical differences were assessed using the Kruskal–Wallis test (*P < 0.05)

The analysis of biomarkers (GFAP, Nfl, t-tau, and UCH-L1) revealed statistically significant differences among the study groups in the initial analysis using Spearman’s correlation (Table 1; Fig. 1).

For GFAP, one-way ANOVA indicated a significant group effect (*p* = 0.006). Multiple comparisons using the Games-Howell test revealed that GFAP levels were significantly higher in the iRBD group compared to the control group (mean difference = 270.68; *p* = 0.037). The PD group did not show a statistically significant difference from the control group for this marker (*p* = 0.467) (Table 2).

**Table 2:** Descriptive profile and variability of biomarkers by group

Biomarker	Control (*n* = 11)	iRBD (*n* = 4)	PD (*n* = 14)	Valor-*p* (ANOVA)
GFAP (ng/mL)	203,19 ± 33,42	473,87 ± 65,97	264,89 ± 39,27	0,006
NFL (pg/mL)	15,18 ± 2,51	25,03 ± 3,26	28,24 ± 3,99	0,037
NFL percentile	56 ± 35	96 ± 3	87 ± 22	0,0162
T-tau (pg/mL)	8,26 ± 0,60	0,79 ± 0,19	1,08 ± 0,13	< 0,001
UCH-L1 (pg/mL)	52,16 ± 5,46	8,32 ± 3,31	9,00 ± 1,14	< 0,001

Values ​​are presented as Mean ± Standard Error of the Mean (SEM). p-value obtained via one-way ANOVA with validation by robust tests (Welch/Brown-Forsythe)

Regarding the Nfl, a significant overall variation was observed (*p* = 0.037), including adjusted values (Table 1). The post hoc test showed that the PD group had significantly higher levels than the control group (28.24 vs. 15.18; *p* = 0.030), whereas the iRBD group, although higher than the control group (25.03), did not reach statistical significance in this comparison (*p* = 0.107) (Table 3).

**Table 3:** Post-hoc multiple comparisons (difference of means)

Pair comparison	GFAP (*p*)	NFL (*p*)	T-tau (*p*)	UCH-L1 (*p*)
Control vs. iRBD	0,037*	0,107*	< 0,001	< 0,001
Control vs. PD	0,467*	0,030*	< 0,001	< 0,001
iRBD vs. PD	0,085*	0,810*	0,432*	0,980*

*p-values ​​were calculated using the Games-Howell test, which is suitable for groups with heterogeneous variances and unequal sample sizes

The biomarkers t-tau and UCH-L1 showed the strongest associations (*p* < 0.001 for both). The Control group showed significantly higher levels of t-tau (8.26 ± 0.60) and UCH-L1 (52.16 ± 5.46) than both pathological groups (*p* < 0.001 in all comparisons with the control). No significant differences were observed between the iRBD and PD groups for these two biomarkers (*p* > 0.40) (Tables 2 and 3).

In our sample, we had 2 patients with antidepressant-related RBD. Their GFAP levels were 105.6 and 121.06 pg/ml; Nfl levels were 5.42 and 7.49 pg/ml (z-scores 0.94 and 0.95) ; t-tau levels were 4.25 and 0.26 pg/ml; UCH-L1 levels were 0.31 and 1.69 pg/ml. These levels are more like those observed in the control group; however, given the limited sample size, no further conclusions can be drawn.

## Discussion

Our study showed that serum levels of GFAP, NfL, t-tau, and UCH-L1 are altered in patients with iRBD compared to an age- and sex-matched control group, with values resembling those observed in individuals with established PD.

The cohort of Zhang et al. evaluated 26 patients with iRBD and found that the baseline plasma Nfl cutoff of 22.93 pg/ml performed best in distinguishing iRBD converters from non-converters [17]. In another cross-sectional study, Teng et al. identified higher levels of GFAP and Nfl in the PD group than in the control group. The cut-offs identified in this previous study were 12.42 for Nfl and 110 for GFAP to identify patients with RBD [18]. In our study, GFAP levels were higher in the iRBD group than in both PD and controls, even after post hoc analysis. This is likely to reflect early astrocytic activation, which may decline in more advanced PD due to predominant neuronal loss. These findings contrast with Youssef et al., possibly due to differences in disease stage among PD participants [10]. In the post-hoc analysis, Nfl levels were statistically higher in the PD group. It remains controversial whether there is a direct relationship between UCH-L1, an essential component of the ubiquitin–proteasome system (UPS), and PD [19, 20].

The study by Nabizadeh et al. evaluated cerebrospinal fluid (CSF) levels of alpha-synuclein (α-syn), beta-amyloid (Aβ1–42), t- tau and p-tau in patients with PD and compared them with healthy controls. These levels were higher in the control group. As with α-syn, reduced Aβ1–42 levels in patients with PD can be explained by greater extracellular accumulation [21]. Consistent with these findings, the t-tau level in peripheral blood was also higher in our control group than in the others.

Another aspect to be considered in the cross-sectional evaluation of biomarkers is the variation that occurs throughout disease progression. The study by Baek et al. analyzed biomarker levels in patients with PD over 3 years. At baseline, patients with PD showed lower CSF α-syn, Aβ, t-tau, and p-tau levels than controls. In all PD patients, CSF α-syn and Aβ decreased in a negative-exponential pattern before the onset of motor symptoms, whereas CSF t-tau, p-tau, and serum NfL, increased [22].

This study has some limitations, including its cross-sectional design, the small sample size, particularly in the iRBD group, and the lack of CSF biomarker data. Peripherally measured t-tau signals can be inflated by non-brain signals. Circulating tau proteins may have extracerebral origins, reducing the marker’s reliability. Nonetheless, the identification of distinct serum biomarker alterations in both iRBD and Parkinson’s disease groups, compared with healthy controls, and the exploratory measurement of specific markers in this context, provides novel pathophysiological insights. Increasing the sample size in future studies will be essential to validate these preliminary findings, improve statistical power, and determine the clinical utility of this biomarker panel for early identification and subtyping of RBD.

## Conclusion

Our study suggested that serum levels of NfL, GFAP, T-tau, and UCH-L1 may serve as potential biomarkers in iRBD, as their profiles closely resemble those observed in patients with PD and differ significantly from those of healthy controls. The GFAP levels performed better in differentiating the iRBD group. These findings may be useful for prognosticating patients at higher risk of developing synucleinopathy. 

## Data Availability

The datasets generated during and/or analysed during the current study are available from the corresponding author on reasonable request.

## Declarations

### Statement regarding the use of AI

AI resources were used solely for language improvement.
